# Type I Interferon Response Is Mediated by NLRX1-cGAS-STING Signaling in Brain Injury

**DOI:** 10.3389/fnmol.2022.852243

**Published:** 2022-02-25

**Authors:** Lauren E. Fritsch, Jing Ju, Erwin Kristobal Gudenschwager Basso, Eman Soliman, Swagatika Paul, Jiang Chen, Alexandra M. Kaloss, Elizabeth A. Kowalski, Taylor C. Tuhy, Rachana Deven Somaiya, Xia Wang, Irving Coy Allen, Michelle H. Theus, Alicia M. Pickrell

**Affiliations:** ^1^Translational Biology, Medicine, and Health Graduate Program, Virginia Polytechnic Institute and State University, Roanoke, VA, United States; ^2^Molecular and Cellular Biology Graduate Program, Virginia Polytechnic Institute and State University, Blacksburg, VA, United States; ^3^Department of Biomedical Sciences and Pathobiology, Virginia Polytechnic Institute and State University, Blacksburg, VA, United States; ^4^Biomedical and Veterinary Sciences Graduate Program, Virginia Polytechnic Institute and State University, Blacksburg, VA, United States; ^5^School of Neuroscience, Virginia Polytechnic Institute and State University, Blacksburg, VA, United States

**Keywords:** brain injury, inflammation, STING, cGAS, innate immunity

## Abstract

**Background:**

Inflammation is a significant contributor to neuronal death and dysfunction following traumatic brain injury (TBI). Recent evidence suggests that interferons may be a key regulator of this response. Our studies evaluated the role of the Cyclic GMP-AMP Synthase-Stimulator of Interferon Genes (cGAS-STING) signaling pathway in a murine model of TBI.

**Methods:**

Male, 8-week old wildtype, *STING* knockout (^−/−^), *cGAS*^−/−^, and *NLRX1*^−/−^ mice were subjected to controlled cortical impact (CCI) or sham injury. Histopathological evaluation of tissue damage was assessed using non-biased stereology, which was complemented by analysis at the mRNA and protein level using qPCR and western blot analysis, respectively.

**Results:**

We found that STING and Type I interferon-stimulated genes were upregulated after CCI injury in a bi-phasic manner and that loss of cGAS or STING conferred neuroprotection concomitant with a blunted inflammatory response at 24 h post-injury. *cGAS*^−/−^ animals showed reduced motor deficits 4 days after injury (dpi), and amelioration of tissue damage was seen in both groups of mice up to 14 dpi. Given that cGAS requires a cytosolic damage- or pathogen-associated molecular pattern (DAMP/PAMP) to prompt downstream STING signaling, we further demonstrate that mitochondrial DNA is present in the cytosol after TBI as one possible trigger for this pathway. Recent reports suggest that the immune modulator NLR containing X1 (NLRX1) may sequester STING during viral infection. Our findings show that NLRX1 may be an additional regulator that functions upstream to regulate the cGAS-STING pathway in the brain.

**Conclusions:**

These findings suggest that the canonical cGAS-STING-mediated Type I interferon signaling axis is a critical component of neural tissue damage following TBI and that mtDNA may be a possible trigger in this response.

## Introduction

Traumatic brain injury (TBI) is a complex neurological condition that is a leading cause of death and disability in children and adults (Roozenbeek et al., 2013; Surgucheva et al., 2014). The injury occurs in two phases: an initial, acute mechanical injury resulting from the external force, and secondary injury/cell death due to complications such as hypoxia, ischemia, and inflammation (Werner and Engelhard, 2007; Greve and Zink, 2009). While the use of improved safety measures has helped minimize the severity of the initial impact, little progress has been made in understanding or treating secondary injuries.

Neuroinflammation is a key mediator of secondary brain injury; however, anti-inflammatory pharmacological approaches largely fail in clinical trials (Simon et al., 2017). Interferons (IFNs) are elevated in post-mortem human TBI samples (IFN-γ; Frugier et al., 2010; Karve et al., 2016) and in experimental TBI murine models (IFN-α, IFN-β, IFN-γ; Lagraoui et al., 2012; Karve et al., 2016), but their functional role has been understudied in TBI. Interferons are produced in response to the detection of pathogen-associated molecular patterns (PAMPs) by pattern recognition receptors (PRRs; Abe et al., 2019). Upon detection of pathogenic nucleic acids, PRRs trigger the production of Type I IFNs to prime both the affected and adjacent cells to neutralize the pathogen. While a number of Type I IFN subtypes have been identified, IFN-α and IFN-β are the most well-studied (Trinchieri, 2010). These IFNs act *via* binding to the cell surface complex known as IFN-α/β receptor (IFNAR), resulting in the expression of IFN-stimulated genes (ISGs) *via* the JAK-STAT pathway (Ivashkiv and Donlin, 2014).

The endoplasmic reticulum protein, Stimulator of Interferon Genes (STING), is known to trigger Type I IFN responses after being activated by cyclic guanosine monophosphate-adenosine monophosphate (cGAMP), a second messenger produced by the DNA sensor, cyclic GMP-AMP synthase (cGAS; Shang et al., 2019; Zhang et al., 2019). cGAS is able to bind nuclear and mitochondrial DNA (Sun et al., 2013; West et al., 2015) to promote STING activation and subsequent translocation of transcription factors (Seth et al., 2005; Ishikawa and Barber, 2008), resulting in the production of innate immune genes, including IFNs and ISGs (Barber, 2014).

Previous studies have demonstrated that STING mRNA is elevated in post-mortem human TBI brain samples, and genetic loss of STING or IFNAR in murine models of TBI reduces lesion size and autophagy markers (Karve et al., 2016; Abdullah et al., 2018). Pharmaceutical inhibition of cGAS, the upstream mediator of STING, in a murine stroke model reduced microglial activation and peripheral immune cell infiltration (Li et al., 2020). Interferon signaling is gaining increasing attention for its role in mediating progressive damage in TBI (Barrett et al., 2020; Sen et al., 2020). Taken together, this suggests that cGAS-STING signaling may represent a novel mechanism for controlling post-traumatic neuroinflammation; however, there is evidence of non-canonical, cGAS-independent STING activation, particularly in response to DNA damage (Dunphy et al., 2018; Unterholzner and Dunphy, 2019). Because upstream STING signaling is undefined in the brain, clarifying the mechanisms of STING activation in the context of inflammation without a known pathogen is critical for identifying targets for therapeutic intervention.

In this study, we utilized genetic knockout mouse models to elucidate the role of the cGAS-STING signaling pathway after TBI in a preclinical model of controlled cortical impact (CCI) injury. We report that the ISG response is immediately upregulated after injury and provide evidence that cytoplasmic mtDNA is available for cGAS binding in the injured cortex. In addition to confirming that loss of endogenous STING is protective (Abdullah et al., 2018), our data suggests that canonical cGAS-STING signaling is a critical component of trauma-induced neuroinflammation and tissue damage. We also uncover *in vivo* evidence for the first time that nucleotide-binding oligomerization domain, leucine-rich repeat containing X1 (NLRX1) abrogates this pathway in the brain. Taken together, we conclude canonical cGAS-STING signaling plays a significant role in influencing TBI outcome.

## Materials and Methods

### Animals

All mice were housed in a pathogen-free facility on a 12 h light/dark cycle at Virginia Tech and provided the standard rodent diet and water *ad libitum*. Male CD-1, C57BL/6J (wildtype), C57/Bl/6J-TMEM173^gt^/J (STING^−/−^; Sauer et al., 2011), and B6(C)-*Cgas*^tm1d(EUCOMM)Hmgu^/J (cGAS^−/−^) mice were purchased from Jackson Laboratories (Ellsworth, ME, USA). *NLRX1*^−/−^ mice were previously described (Allen et al., 2011). *STING*^−/−^, cGAS^−/−^, and *NLRX1*^−/−^ mice were genotyped according to protocols provided by Jackson Laboratories. All experiments were conducted in accordance with the NIH Guide for the Care and Use of Laboratory Animals and under the approval of the Virginia Tech Institutional Animal Care and Use Committee.

### Controlled Cortical Impact (CCI) Injury

Animals were prepared for surgery as previously described (Brickler et al., 2016). Male CD-1, wildtype, STING^−/−^, cGAS^−/−^, and NLRX1^−/−^ mice aged 8–10 weeks were anesthetized with an intraperitoneal injection of ketamine (100 mg/kg) and xylazine (10 mg/kg), then positioned in a stereotactic frame. Body temperature was continually monitored *via* a rectal probe and maintained at 37°C with an autoregulated heating pad. A 4 mm craniotomy was made with a portable drill over the right parietal-temporal cortex (−2.5 mm A/P and 2.0 mm lateral from bregma). Moderate CCI was induced with an eCCI-6.3 device (Custom Design and Fabrication, Richmond, VA, USA) using a 3 mm impact tip at an angle of 70°, 5.0 m/s velocity, 2.0 mm impact depth, and 100 ms dwell period (Theus et al., 2010). The incision was closed with Vetbond tissue adhesive (3M, St. Paul, MN, USA), and post-surgery animals received Buprenorphine SR (1 mg/kg, ZooPharm, Windsor, CO, USA) subcutaneously. Sham animals received a craniotomy only.

### Histology and TUNEL Staining

At the indicated times post-CCI injury, mice were anesthetized by isoflurane (IsoFlo^®^, Zoetis, Parsippany-Troy Hills, NJ, USA) and euthanized by cervical dislocation. Brains were fresh frozen on dry ice while embedded in O.C.T. (Tissue-Plus^TM^ O.C.T. Compound, Fisher HealthCare, Houston, TX, USA). Brains were coronally sectioned (30 μm thickness) using a cryostat (CryoStar NX50, Thermo Scientific, Waltham, MA, USA) through the lesion site (−1.1 to −2.6 mm posterior to bregma). Serial sections 300 μm apart were stained with Cresyl violet (Electron Microscopy Sciences, Hatfield, PA, USA).

To identify cells undergoing apoptosis, slides were fixed in 10% formalin (Fisher Chemicals, Pittsburgh, PA) for 5 min, washed with 1× PBS, permeabilized in 2:1 ethanol:acetic acid at −20°C for 10 min and 0.4% Triton for 5 min, then washed with 1× PBS and TUNEL stained according to the manufacturer’s suggestions (DeadEnd^TM^ Fluorometric TUNEL System, Promega, Madison, WI). Slides were then fixed for 5 min in 10% formalin, blocked for 30 min in 0.2% Triton, 2% cold water fish gelatin (Sigma, St. Louis, MO, USA), and stained for Nissl (1:100, NeuroTrace^TM^ 530/615 Red Fluorescence Nissl, Invitrogen, Carlsbad, CA, USA). Slides were mounted with DAPI Fluoromount-G (SouthernBiotech, Birmingham, AL, USA). Representative confocal images were taken on a Nikon C2 at 20× magnification using the recommended *z*-step size. Maximum intensity projections were created in Nikon NIS-Elements.

### Estimating Lesion Size and TUNEL^+^/Nissl^+^ Cells

Lesion volume (mm^3^) was assessed by a blinded investigator using StereoInvestigator’s Cavalieri estimator (MicroBrightField, Williston, VT, USA) and an Olympus BX51TRF motorized microscope (Olympus America, Center Valley, PA, USA), as previously described (Theus et al., 2017). Five coronal serial sections for each animal were spaced 300 μm apart surrounding the epicenter of injury were stained for Nissl (described above) and viewed at 4× magnification under brightfield illumination. A grid (100 μm spacing) was set over the ipsilateral lesion site and markers were placed over the contused tissue, as identified by diminished Nissl staining intensity, morphology, and pyknotic neurons. The contoured area with the section thickness, section interval, and the number of sections were used by the Cavalieri program to estimate the volume of contused tissue.

Apoptotic cells (TUNEL^+^) were counted by a blinded investigator using five adjacent coronal serial sections (spaced 300 μm apart) with the StereoInvestigator Optical Fractionator (MicroBrightField, Williston, VT, USA) probe. Approximately 100 randomized sites per animal (grid size: 500 × 500 μm, counting frame size: 100 × 100 μm) were assessed to identify TUNEL^+^ and TUNEL^+^/Nissl^+^ cells (apoptotic neurons), and section thickness was estimated every five sites to improve the accuracy of the cell count estimation. The number of cells per contour, average estimated section thickness, section interval, and the number of sections were used to estimate the number of cells within the lesion volume.

### Real Time qPCR

A 4 × 4 mm section of the injured cortex tissue was micro-dissected from each animal and immediately submerged in TRIzol^TM^ Reagent (Invitrogen, Carlsbad, CA, USA). Either sham surgery animals’ parietal cortices or the contralateral parietal cortex from injured animals were extracted to serve as the control. Cortical tissue was mechanically homogenized, lysed, and extracted with TRIzol^TM^ Reagent (Invitrogen, Carlsbad, CA, USA) following the manufacturer’s protocol. RNA was reverse transcribed to cDNA using iScript cDNA Synthesis Kit (Bio-Rad, Hercules, CA, USA). Reactions containing SYBR Green PCR Master Mix (Bio-Rad, Hercules, CA, USA), 10–50 ng of cDNA, and 0.4 mM of each primer set were run on the CFX96 System (Bio-Rad, Hercules, CA, USA). qPCRs were performed in technical triplicates for each gene/primer set (Table 1). Expression levels were normalized to GAPDH and fold change was determined by the comparative C_T_ method (Schmittgen and Livak, 2008). Primer efficiency was determined using a 4-point log concentration curve (Bio-Rad CFX Maestro software, Hercules, CA, USA).

**Table 1:** qPCR primers used in experiments.

Gene	Forward Seq. (5′ - 3′)	Reverse Seq. (5′ - 3′)
IRF7	CAA TTC AGG GGA TCC AGT TG	AGC ATT GCT GAG GCT CAC TT
IFIT1	ACC ATG GGA GAG AAT GCT GAT G	TGT GCA TCC CCA ATG GGT TC
STAT1	GCG GCA TGC AAC TGG CAT ATA ACT	ATG CTT CCG TTC CCA CGT AGA CTT
STAT2	TGA TCT CTA ACA GAC AGG TGG	CTG CAT TCA CTT CTA AAG ACT C
IFIT3	ATC ATG ATG GAG GTC AAC CG	TTG CAC ACC CTG TCT TCC AT
IFNA4	CTT TCC TCA TGA TCC TGG TAA TGA T	AAT CCA AAA TCC TTC CTG TCC TCC
IFNB1	AAC TCC ACC AGC AGA CAG TG	GGT ACC TTT GCA CCC TCC AG
RIG-I	GAG TAC CAC TTA AAG CCA GAG	AAT CCA TTT CTT CAG AGC ATC C
IFIH1	CGG AAG TTG GAG TCA AAG C	TTT GTT CAG TCT GAG TCA TGG
IL-10	AGA CCA AGG TGT CTA CAA GGC	TCA TCA TGT ATG CTT CTA TGC AGT
IL-6	ACA AGT CGG AGG CTT AAT TAC ACA	TTG CCA TTG CAC AAC TCT TTT C
MCP1	TCA CCT GCT GCT ACT CAT TCA CCA	TAC AGC TTC TTT GGG ACA CCT GCT
CXCL10	ATA ACC CCT TGG GAA GAT GGT G	CTA GCT CAG GCT CGT CAG TTC
GAPDH	ATT GTG TCC GTC GTG GAT CTG A	AGA TGC CTG CTT CAC CAC CTT CTT
STING	GCC TTC AGA GCT TGA CTC CA	GTA CAG TCT TCG GCT CCC TG
ND1	CAG CCT GAC CCA TAG CCA TA	ATT CTC CTT CTG TCA GGT CGA A
COX1	AGG CTT CAC CCT AGA TGA CAC	GTA GCG TCG TGG TAT TCC TGA A

*List of forward and reverse sequences used for qPCR*.

### Western Blot

A 4 × 4 mm section of injured cortex tissue was micro-dissected from each animal, snap- frozen in liquid nitrogen, and stored at −80^o^C until use. Extracts were homogenized with a hand-held mortar/pestle (VWR, Radnor, PA, USA) on ice in RIPA buffer (Thermo Scientific Pierce Protein Biology, Waltham, MA, USA) containing proteinase and phosphatase inhibitors (Thermo Scientific Pierce Protein Biology, Waltham, MA, USA). Homogenates were spun at 4^o^C at 15,000× *g* for 15 min and the supernatant was stored at −80^o^C until use. Protein quantification was determined using the DC protein assay kit with BSA standards (Bio-Rad, Hercules, CA, USA). Fifty milligram of protein was run on a 4–12 percent NuPage Bis-Tris Gel (Thermo Fisher Scientific, Waltham, MA, USA) and transferred onto a PVDF membrane (MilliporeSigma, Burlington, MA, USA). Primary antibodies were incubated overnight. Primary antibodies used were p-STING S365, STING, cGAS, histone H3 (Cell Signaling Technology, Danvers, MA, USA), α-tubulin (MilliporeSigma, Burlington, MA, USA), Mfn2 (was a kind gift from Richard Youle’s laboratory), and HMGB1 (R&D Systems, Minneapolis, MN, USA). Membranes were washed in 1× TBST, and secondary HRP conjugated antibodies (Jackson ImmunoResearch Laboratories, Inc., West Grove, PA, USA) were incubated at RT for 1 h. Chemiluminescent detection (Thermo Scientific Pierce Protein Biology, Waltham, MA, USA) was used to detect a signal with the Bio-Rad ChemiDoc system (Bio-Rad, Hercules, CA, USA). Relative optical density was determined with ImageLab software (Bio-Rad, Hercules, CA, USA).

### Evans Blue

Twenty-four hours after the CCI injury, the animals received an intravenous injection of 300 μl Evans blue. After 3 h, animals were sacrificed, and ipsilateral and contralateral hemispheres were collected. The distribution of Evans Blue was verified by opening the thoracic and abdominal cavities. The tissue was incubated in 500 μl 10% formamide at 55°C for 24 h, then centrifuged for 4 min at 210× *g* to pellet the tissue. Absorbance for each hemisphere was measured in triplicate at 610 nm.

### Rotarod

Gross motor function was evaluated by Rotarod (Columbus Instruments, Columbus, OH, USA) testing from 4 to 14 days post-TBI. The initial velocity was 5 rpm, with an acceleration of 0.1 rpm/s. Each animal underwent three trials per day with a 2 min rest between each trial. The average time of the three trials was used for analysis. Eight-week-old animals were trained for four consecutive days with a baseline measurement taken on the 5th day. Animals underwent sham or CCI surgery, then rotarod performance was evaluated at 4-, 7-, and 14-days post-surgery. Each animal’s performance was compared to its baseline measurement, and average performance for all animals was reported. After the final day of testing, animals were euthanized for histology, qPCR, or western blotting as described above.

### Cytosolic Fractionation

The cytosolic fraction was extracted as previously reported (West et al., 2015). Cortical tissue was homogenized in PBS plus protease and phosphatase inhibitors (Thermo Scientific Pierce Protein Biology, Waltham, MA, USA). Dissociated tissues were incubated in the cytosolic extraction buffer containing 150 mM NaCl, 50 mM HEPES, pH 7.4, and 15–25 μg/ml digitonin (Gold Biotechnology, St Louis, MO, USA). The homogenates were incubated end over end for 10 min to allow selective plasma membrane permeabilization, then centrifuged at 980 *g* for 3 min three times to pellet intact cells. Pellets were retained for western blotting. The supernatant was centrifuged at 17,000 *g* for 10 min to pellet any remaining cellular debris. DNA was extracted the Zymo DNA extraction kit.

MtDNA detection and quantification of cytosolic extracts were performed by real time PCR as previously published (West et al., 2015). Ct values obtained for mtDNA abundance for whole cell extracts served as normalization controls for the mtDNA values obtained from the cytosolic fractions. It was also used to ensure there were no hemispheric differences in mtDNA levels in an individual animal. To prepare extracts, injured and contralateral tissues were weighed, and 25 mg of tissue was used for downstream processing. Ten percent of the homogenized tissue was sampled prior to cytosolic fractionation for whole cell extracts. Ct values from the ipsilateral cortex were normalized to that individual animal’s contralateral hemisphere using the comparative Ct method as whole mtDNA copy number as the reference.

### Cell Isolations

Murine cells were isolated using the Worthington Dissociation Kit (Worthington Biochemical Corporation, Lakewood, NJ, USA) and slight modifications to published protocols (Holt and Olsen, 2016; Holt et al., 2019). Briefly, WT animals were deeply anesthetized with a ketamine (500 mg/kg)/xylazine (10 mg/kg) cocktail and hand perfused with cold PBS to remove blood. The brain was removed, cortices dissected, and finely minced in warmed papain with DNase. Tissue was digested in papain at 37°C for 15 mn for astrocytes and endothelial cells or 45 min for microglia with gentle inversions every 5 min. For astrocytes and endothelial cells, the solution was triturated, centrifuged at 300 *g* for 5 min 4°C, and the pellet was resuspended in resuspension buffer per the Worthington protocol to stop the digestion. The dissociated cells were spun down again, filtered through a 70 μm cell strained with 10 ml 0.5% BSA PBS, then resuspended in 200 μl 0.5% BSA PBS and microbeads. Oligodendrocytes were removed with anti-myelin beads, then endothelial cells and astrocytes were isolated with CD31 and ACSA-2 beads (all microbeads from Miltenyi Biotec, Auburn, CA, USA), respectively, per published protocols (Holt and Olsen, 2016; Holt et al., 2019). *Microglia*: Microglia were isolated by plating the cells collected following the Worthington Papain Dissociation System protocol. Cells were incubated for one hat 37°C and non-adherent cells were washed off, leaving microglia adherent to the plate. *Primary Neurons*: Primary neurons were isolated from P0 mouse pups per the Worthington Papain Dissociation System protocol and cultured on poly-d-lysine-coated plates in Neurobasal Medium with B27 supplement (Gibco, Waltham, MA, USA). Primary neurons were collected 14 days after plating for RNA isolation.

### Statistical Analysis

Data were analyzed with GraphPad Prism 9 (GraphPad, San Diego, CA, USA). A Student’s two-tailed *t*-test was used for comparison of two experimental groups. One-way or two-way ANOVA with Tukey’s multiple comparison test were used for comparison of more than two experimental groups as appropriate. Differences were considered statistically significant at *p* < 0.05. Data reported as mean ± SEM. *n* values are reported in the figure legends.

## Results

### CCI Injury Induces a Biphasic ISG Response in the Damaged Cortex

**Figure 1:**
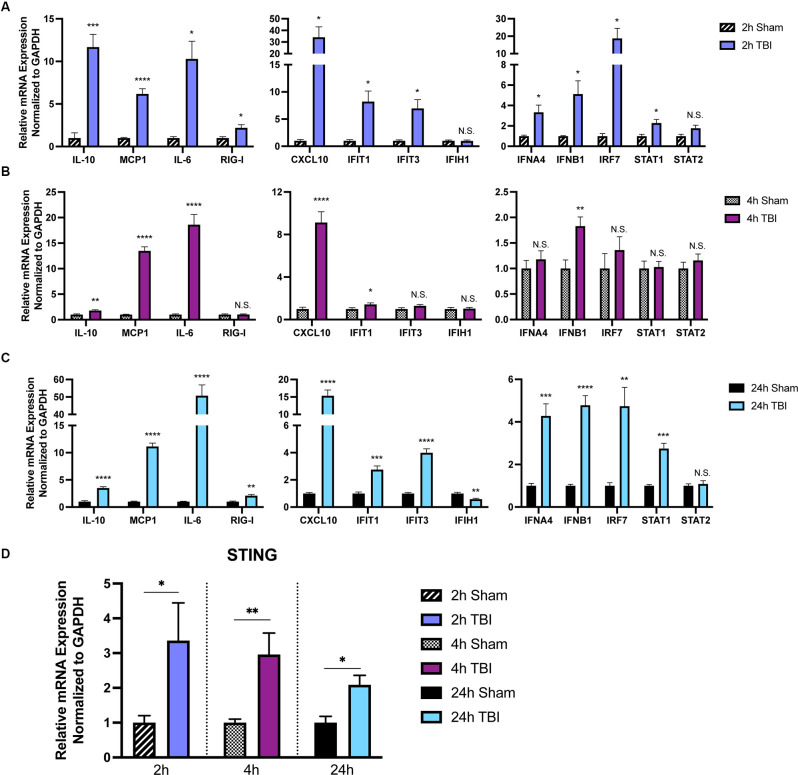
The immune response to TBI is biphasic. Cytokine and interferon-stimulated gene (ISG) expression profiled at 2 h **(A)**, 4 h **(B)**, and 24 h **(C)** after injury or sham surgery in male CD-1 mice. **(D)** mRNA expression of STING in the ipsilateral cortex 2, 4, and 24 h after injury or sham surgery. Gene expression was normalized to GAPDH. *n* = 5–7 per group. Data presented as mean ± SEM. *p < 0.05, **p < 0.01, ***p < 0.001, ****p < 0.0001. N.S. = not significant.

To provide further insight into how TBI alters inflammatory gene transcription in a temporal manner, we first sought to broadly profile changes in cytokines, PRRs, ISGs, IFNs, and transcription factors that are known to be upregulated by the innate immune system (Schneider et al., 2014). Cortices from male 8-week injured mice showed a temporally biphasic increase in mRNA expression for most (10 of 13) genes tested compared to shams (Figures 1A–C). Expression of *Il-10, MCP-1, RIG-I, CXCL10, IFIT1, IFIT3, IFNA4, IFNB1, IRF7*, and *STAT1* was significantly increased at 2- and 24-h (h) post-injury, which was blunted at 4 h. *IFIH1* (also known as MDA5), and *STAT2* expression were unchanged. Furthermore, *Il10, MCP1*, and *Il-6* did not show a biphasic expression pattern. Of note, the Type I IFNs *IFNA4* and *IFNB1* showed a biphasic upregulation after injury.

Previous reports demonstrate neuroprotection in *STING*^−/−^ mice after CCI injury (Abdullah et al., 2018). To gain a more in-depth understanding of the expression pattern of STING, we assessed mRNA levels at 2, 4, and 24 h in the ipsilateral parietal cortex (Figure 1D). We find *STING* is upregulated at all time points tested but shows the greatest change in expression at 2 and 4 post-injury (Figure 1D). Interestingly, STING itself is an ISG and is positively regulated by its own transcription upon activation (Ma et al., 2015). Taken together, these data demonstrate a strong innate immune response occurring within hours after TBI.

### CCI Injury Induces the Presence of Cytosolic Mitochondrial DNA in Damaged Cortex

Loss of STING (Abdullah et al., 2018), IFNAR (Karve et al., 2016), or IFNβ (Barrett et al., 2020; Sen et al., 2020) function has been shown to be beneficial in TBI outcome; however, the mechanism regulating their induction remains unclear. The canonical STING-cGAS pathway is activated by the binding of viral nucleic acids found in the cytoplasm (Sun et al., 2013), resulting in the production of the second messenger cGAMP which binds and activates STING (Shang et al., 2019; Zhang et al., 2019). In addition, mitochondrial DNA (mtDNA) can activate STING in models where mtDNA packaging proteins and mitochondrial permeability proteins are disrupted genetically (West et al., 2015; McArthur et al., 2018), and mtDNA is present in cerebral spinal fluid and serum following TBI (Walko et al., 2014; Kilbaugh et al., 2015).

**Figure 2:**
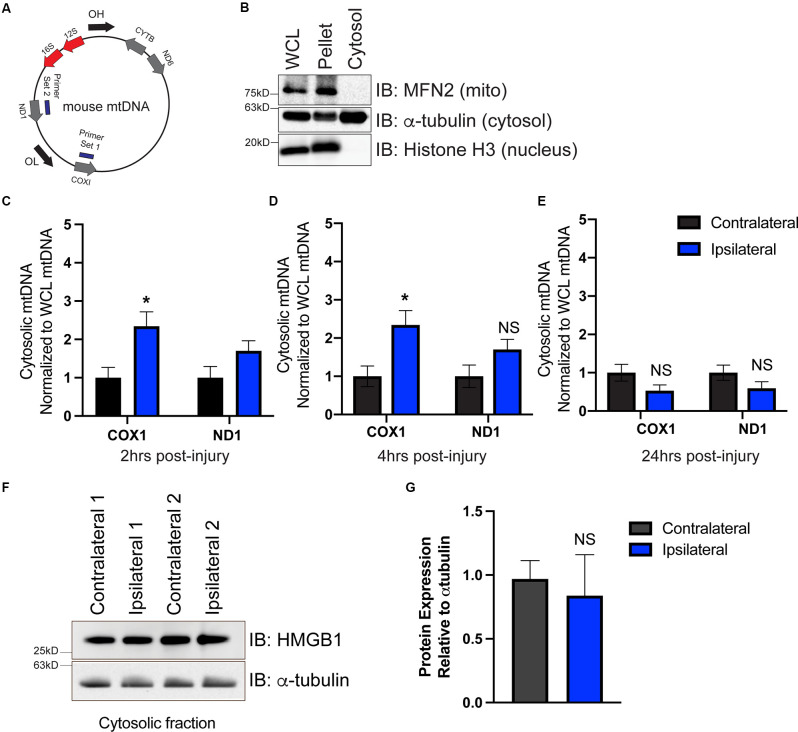
mtDNA is present in the cytosol as a possible trigger for cGAS-STING activation after TBI. **(A)** Diagram of mtDNA showing primer sets for ND1 and COX1. **(B)** Western blot of whole cell lysate (WCL), cell pellet, and isolated cytosol to confirm cytosolic purity. Mitofusin 2 (MFN2) was used to identify mitochondria, alpha-tubulin was used for detecting cytosol, and histone H3 indicated the nuclear fraction. **(C–E)** mtDNA was directly detected in the cytosol *via* qPCR at 2, 4, and 24 h post-TBI. Each animal was normalized to the respective hemisphere’s WCL. **(F)** Representative western blotting of the DNA-binding protein high mobility group box protein 1 (HMGB1) in the cytosol 2 h post-TBI. **(G)** Quantification of HMGB1 western blot normalized to alpha-tubulin. *n* = 5 per group for all experiments. Data presented as mean ± SEM. *p < 0.05. NS = not significant.

To determine whether mtDNA is present in the cytoplasm, we isolated the cytoplasmic fraction of cells isolated from the ipsilateral and contralateral cortex. We used primers that targeted two different locations on the mitochondrial genome corresponding to the coding region for COX1 and ND1 (Figure 2A). To ensure that our cytosolic fractions were enriched, western blotting detected the presence of the cytosolic protein α-tubulin but showed that the cytosolic fraction was devoid of the nuclear protein histone H3 and the outer mitochondrial membrane protein Mfn2 (Figure 2B). Interestingly, we saw a significant elevation in mtDNA at 2 h (Figure 2C), and 4 h (Figure 2D) post-injury, which was resolved by 24 h (Figure 2E), indicating that mtDNA is present in the cytoplasm of the injured cells. These data correlated with ISG induction at 2 h post-injury (Figure 1A).

To determine whether cytoplasmic nuclear DNA was also present, we performed western blotting on cytoplasmic extracts at 2 h to evaluate the expression of the chromosome-associated protein nuclear protein high mobility group box protein 1 (HMGB1), whose expression is increased when nuclear DNA is present in the cytosol (Urbonaviciute et al., 2008; Pisetsky, 2014). cGAS is also more easily bound to and activated by HMGB1-coated nuclear DNA than DNA in its free form (Andreeva et al., 2017). We found HMGB1 protein was present in cytosolic fractions isolated from both contralateral and ipsilateral hemispheres (Figure 2F); however, ipsilateral cytoplasmic HMGB1 expression was not increased compared to contralateral (Figure 2G). This suggests that mtDNA is available to drive cGAS activation in the damaged cortex after CCI injury, though alternative DNA sources (including nuclear DNA) cannot be fully ruled out.

### Loss of cGAS-STING Confers Neuroprotection After CCI Injury

**Figure 3:**
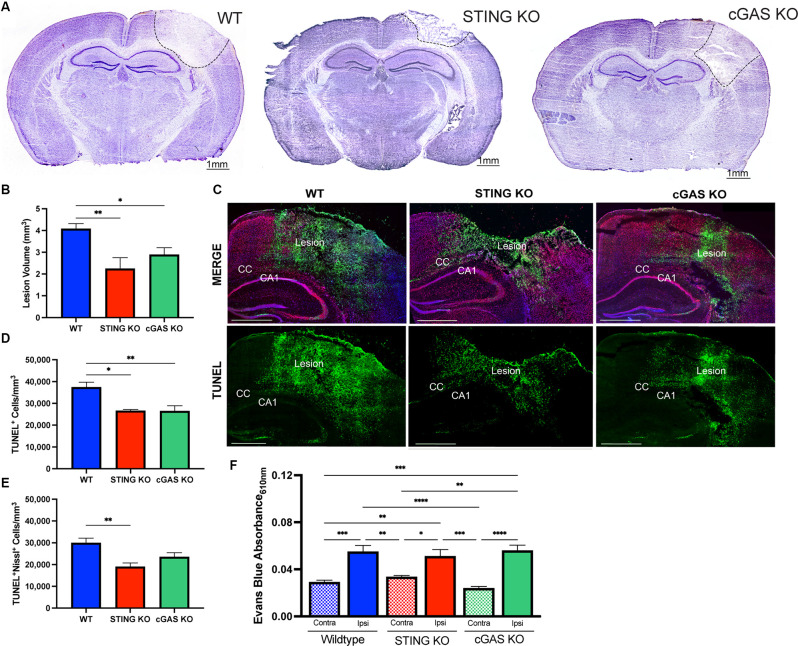
Loss of endogenous cGAS and STING decreases lesion volume and cell death after TBI. **(A)** Representative images of Cresyl violet stained brains at 4x magnification. The dashed line indicates the lesion site. Scale bar = 1 mm. **(B)** Quantification of lesion volume in Cresyl violet stained WT, *STING^−/−^*, and *cGAS*^−/−^ brains 24 h after CCI injury. *n* = 6–11 per genotype. **(C)** Representative confocal images of TUNEL (green), Nissl (red), and DAPI (blue) at 20x magnification of ipsilateral hemisphere of CCI-injured WT, STING^−/−^, and cGAS^−/−^ animals. Scale bar = 1 mm. CC = corpus callosum. CA1 = hippocampal cornu ammonis 1. **(D)** Quantification of the density of apoptotic cells (indicated by cells labeled with TUNEL per mm^3^) in the lesion site 24 h after injury in WT, *STING*^−/−^, and *cGAS*^−/−^ mice. **(E)** The density of apoptotic neurons (indicated by cells positive for both TUNEL and Nissl per mm^3^) in the lesion site 24 h after injury in WT, STING^−/−^, and *cGAS*^−/−^ mice. *n* = 6–12 per group. **(F)** Quantification of Evans blue absorbance (O.D. 610 nm) from contralateral and ipsilateral cortex 24 h after injury in WT, *cGAS*^−/−^, and *STING*^−/−^ mice. *n* = 6–7 per genotype. Data presented as mean ± SEM. *p < 0.05, **p < 0.01, ***p < 0.001, ****p < 0.0001.

cGAS is necessary for canonical STING activation (Sun et al., 2013; Wu et al., 2013). To verify this pathway’s involvement in TBI, we utilized cGAS KO mice (*cGAS*^−/−^; Supplementary Figure 1A) and STING KO (*STING*^−/−^; Supplementary Figure 1B) mice. *STING*^−/−^ mice displayed a significant reduction in lesion volume compared to WT at 1 day post-injury (dpi; Figures 3A,B), confirming prior work (Abdullah et al., 2018). Moreover, *cGAS*^−/−^ mice also showed significant neuroprotection compared to WT mice (Figures 3A,B). To determine whether the reduction in lesion volume was due to increased neuronal survival, we performed immunodetection of apoptotic neurons by TUNEL staining (Figure 3C). TUNEL detects nuclear DNA fragmentation, a hallmark of apoptosis and necrosis (Grasl-Kraupp et al., 1995). A significant reduction of TUNEL^+^ cells was detected 24 h after injury in both *cGAS*^−/−^ and *STING*^−/−^ mice (Figure 3D). Co-labeling with Nissl, an unspecific neuronal marker, showed the number of apoptotic neurons was significantly reduced in the ipsilateral cortex of *STING*^−/−^ mice after injury and trending toward a significant reduction in *cGAS*^−/−^ mice (Figure 3E). Although cGAS/STING deficiency is neuroprotective, no difference was observed in blood-brain barrier function as seen by quantifying Evans Blue infiltration in the damaged cortex compared to contralateral (Figure 3F). Our results suggest that the cGAS-STING pathway contributes to the neurotoxic effects induced by CCI injury.

Behavioral impairments have been previously assessed in *IFNβ^−/−^* mice after TBI (Barrett et al., 2020), therefore we sought to provide further confirmation that canonical cGAS-STING signaling is critical in TBI outcome. Using rotarod assessment, we found no difference in motor function between sham-injured *cGAS*^−/−^ and WT mice (Supplementary Figure 2A). However, *cGAS*^−/−^ mice showed a significant reduction in motor deficit at 4 dpi compared to WT (Supplementary Figure 2B) but no difference at 7 and 14 dpi (Supplementary Figure 2B). *cGAS*^−/−^ mice also showed a significant reduction in lesion volume at 14 dpi relative to WT (Supplementary Figures 2C,D), despite their comparable motor performance (Supplementary Figure 2B). Similarly, *STING*^−/−^ mice also showed reduced lesion volume at 14 dpi (Supplementary Figures 2C,D). We also assessed mRNA levels of *IFNA4*, *IFNB1*, and *IL6* at 14 days post-injury. Interestingly, all three genes were downregulated at this chronic timepoint relative to WT sham animals (Supplementary Figure 2E).

### Loss of cGAS-STING Ameliorates Pro-inflammatory Gene Expression After CCI Injury

**Figure 4:**
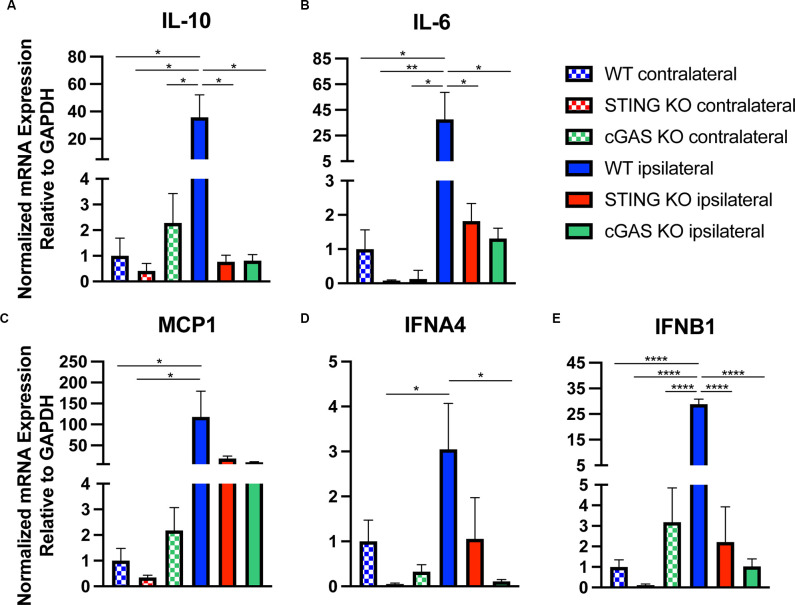
Loss of cGAS-STING blunts the innate immune response to TBI. Quantified mRNA expression of **(A)** *Il10*, **(B)** *Il6*, **(C)** *MCP1*, **(D)** *IFNA4*, and **(E)** *IFNB1* assessed by qPCR at 24 h post-injury in wildtype, *STING*^−/−^, and *cGAS*^−/−^ cortices. Gene expression normalized to GAPDH. *n* = 5–6 per group. Data presented as mean ± SEM. *p < 0.05, **p < 0.01, ****p < 0.0001.

In addition to histological and functional changes, we profiled changes in gene expression in the cortex at 24 h post-injury in WT, *STING^−/−^*, and *cGAS*^−/−^ mice. We found no difference in the contralateral cortex when compared to sham (Supplementary Figure 3), therefore we used contralateral tissue when performing our relative analysis. Both *STING*^−/−^, and *cGAS*^−/−^ mice showed a significant reduction in mRNA expression of *Il10, Il6, MCP1, IFNA4*, and *IFNB1* (Figures 4A–E) in the ipsilateral cortex when compared to WT. To provide further insight into the transcriptional changes, we assessed the complete panel of genes described in Figure 1. We found all genes tested were significantly altered in *STING*^−/−^ mice compared to WT (Supplementary Figure 4). These findings suggest that cGAS-STING signaling plays a key role in regulating innate immune gene expression in the damaged cortex after CCI injury.

### Microglia Are the Predominant Cell Type Expressing cGAS and STING in the Brain

**Figure 5:**
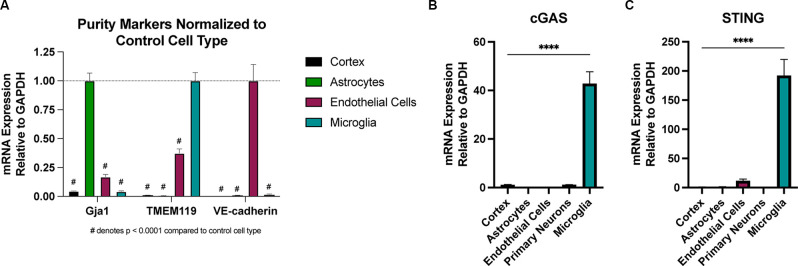
Microglia are the predominant cell type expressing cGAS and STING in the CNS. Cell type specific populations were isolated from naïve (uninjured) WT male animals. **(A)** mRNA expression of gap junction associated protein 1 (*Gja1*), transmembrane protein 119 (*TMEM119*), and vascular-endothelial cadherin (*VE-cadherin*), genes characteristic of astrocytes, microglia, and endothelial cells, respectively. Data is normalized to the characteristic (control) cell type to show the purity of isolated populations. ^#^*p* < 0.0001 compared to control cell type. **(B)** *cGAS* and **(C)** *STING* mRNA expression in isolated cell types and whole cortex. *n* = 3–5 per cell type. ****p < 0.0001.

There is conflicting evidence regarding which CNS cell types express cGAS and STING (Nazmi et al., 2012; Abdullah et al., 2018; Li et al., 2020; Zhang et al., 2020). To test this, we employed several techniques for isolating pure CNS cell populations for qPCR assessment. Naïve (uninjured) astrocytes and endothelial cells were extracted using magnetic bead sorting (Holt and Olsen, 2016; Holt et al., 2019), while the remaining cells were plated for isolating microglia and primary neuronal cultures to assess expression in these cell types. Real-time qPCR analysis of cell-type-specific genes was used to verify the purity of the isolated cell populations (Figure 5A). We observed that naïve microglia showed the greatest enrichment of transcripts for both cGAS and STING when compared to all other cell types (Figures 5B,C). In line with the biphasic cytokine response we noted from the whole cortex after injury, microglia showed upregulation of cGAS at 24 h post-injury and a trending increase of STING at 2- and 24 h after CCI (Supplementary Figures 5A,B). Non-adherent cells (CNS cells remaining after the removal of microglia) did not show increased expression of STING in response to injury. cGAS expression in these cells was undetectable in the sham groups making it difficult to quantify (data not shown). This suggests that microglia are a main cell type influencing the Type I interferon response *via* the cGAS-STING pathway in TBI.

### NLRX1 Negatively Regulates cGAS-STING Activation After CCI Injury

**Figure 6:**
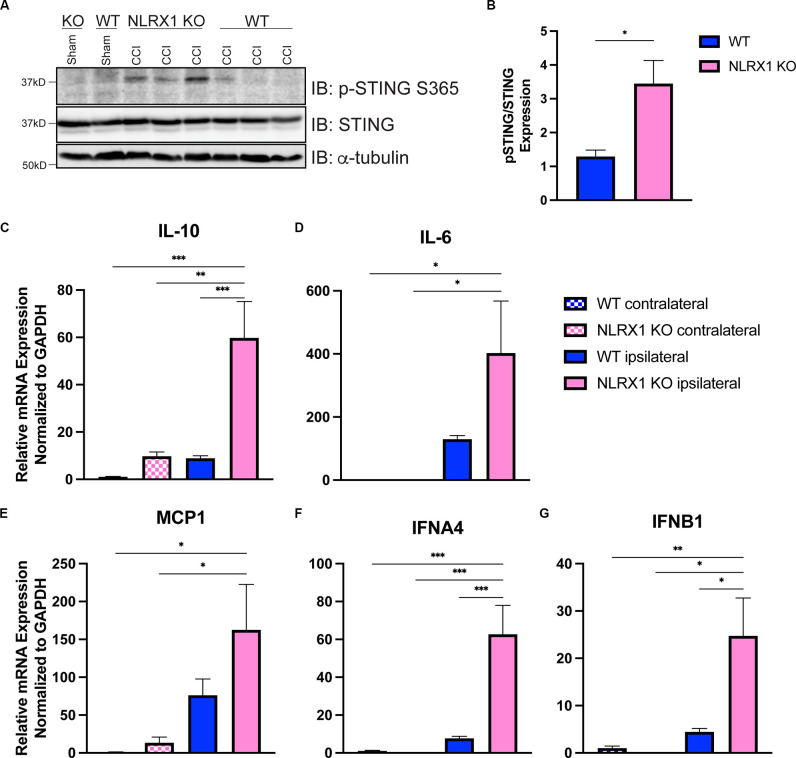
NLRX1 negatively restricts cGAS-STING activation after injury. **(A)** Representative western blotting of phosphorylated STING, STING, and alpha tubulin in WT and NLRX1 KO cortical tissue 3 days post-injury or sham surgery. **(B)** Quantification of pSTING/STING, normalized to alpha tubulin shown in **(A)**. mRNA expression of **(C)** *Il10*, **(D)** *Il6*, **(E)** *MCP1*, **(F)** *IFNA4*, and **(G)** *IFNB1* assessed by qPCR at 24 h post-injury in wildtype and *NLRX1*^−/−^ cortices. Gene expression normalized to GAPDH. *n* = 5–7 per group. Data presented as mean ± SEM. *p < 0.05, **p < 0.01, ***p < 0.001.

We recently showed that loss of NLRX1 exacerbates tissue damage after CCI injury, in part by increasing NF-κB activity in microglial and/or peripheral-derived immune cells (Theus et al., 2017). It is also well-established that NLRX1 may sequester STING to prevent the interferon response (Guo et al., 2016); however, this association has not been evaluated in the brain. To test whether NLRX1 represents a novel upstream regulator of STING in the cortex after injury, we evaluated activated STING expression and the ISG response. Interestingly, *NLRX1^−/−^* mice showed a significant increase in activated (phosphorylated) *p*-STING (S365) compared to WT at 3 dpi (Figures 6A,B). We also assessed mRNA expression of *IL10, IL6, MCP1, IFNA4*, and *IFNB1* 24 h post-injury. Relative to WT, *NLRX1*^−/−^ mice showed a significant increase in cortical expression of all genes tested, including the interferons *IFNA4* and *IFNB1* (Figures 6C–G). These data suggest that NLRX1 plays a central role in suppressing the Type I interferon response by limiting STING activation following CCI injury.

## Discussion

**Figure 7:**
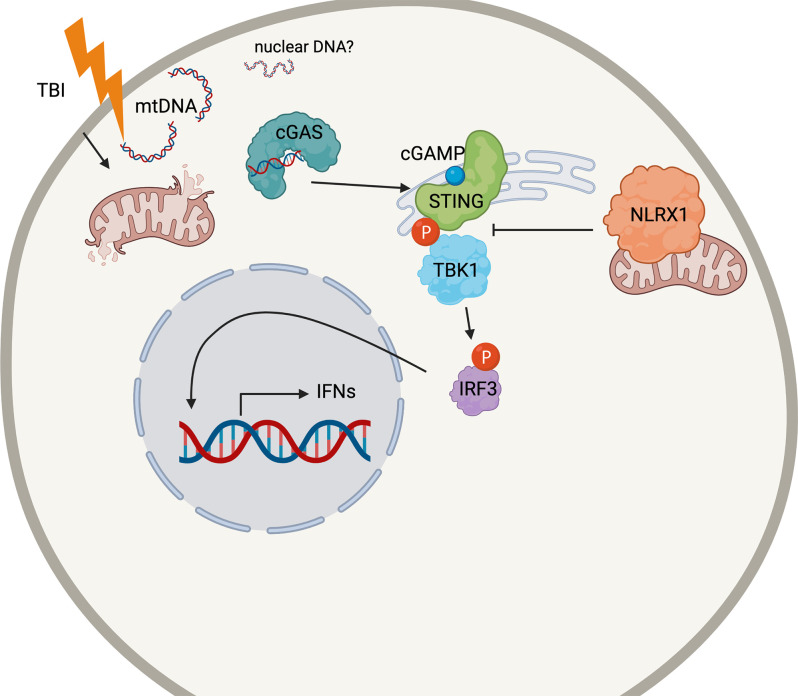
Schematic cartoon of the proposed mechanism. Following TBI, mitochondria release DNA (mtDNA) into the cytoplasm which may bind cGAS to prompt STING activation. STING is phosphorylated by TBK1, which also phosphorylates the transcription factor IRF3. Nuclear translation of IRF4 causes the production of the Type I interferon response in the injured brain. NLRX1 may sequester STING to block its phosphorylation by TBK1. The figure was made with BioRender.com.

Our data suggest that the antiviral interferon pathway mediated by cGAS-STING contributes to the secondary injury after TBI (Figure 7). The activation of STING in the nervous system has recently been brought to the attention of those studying CNS viral infections. STING is highly conserved among organisms (Goto et al., 2018; Martin et al., 2018) and restricts Zika infection in the *Drosophila* brain (Liu et al., 2018). Microglial expression and activation of STING also restricts herpes simplex virus-1 (HSV-1) infection in neurons or promotes apoptosis, depending on viral load (Reinert et al., 2016, 2021). However, the classical viral/microbe-induced innate immune pathways in the brain may not necessarily need viral-induced stimulation for activation. In mouse models of multiple sclerosis, a demyelinating neurodegenerative disease, STING may control microglial reactivity (Mathur et al., 2017).

The present work demonstrates that STING is upregulated in the ipsilateral cortex of CCI-injured mice, which correlates with a biphasic increase in a variety of cytokines, including *IFNA4* and *IFNB1*. We show that cGAS and STING are highly expressed in microglia (Figure 5) and that microglia show increases in expression of cGAS 24 h after injury (Supplementary Figure 5), suggesting they may be the primary cell type responsible for this response. Of note, it is unclear whether neurons contribute to cGAS-STING signaling after TBI. While our data show that STING expression in primary neurons is low (Figure 5), we were unable to isolate healthy neurons from adult animals in a similar manner to the other cell types due to neuronal breakage; therefore, it is possible that mature neurons *in vivo* have higher baseline STING expression. We also collected the non-microglial population of cells for qPCR analysis when isolating microglia. These cells did not show cGAS or STING expression (Supplementary Figure 5C); however, it is possible that very few neurons survived the dissociation process. Regardless, our data suggest that microglia are likely the predominant cell type driving cGAS-STING signaling after TBI.

While previous work has shown that loss of endogenous STING reduces lesion size following TBI (Abdullah et al., 2018), recent evidence suggests that STING may be able to function independently of its canonical upstream mediator, cGAS (Almine et al., 2017; Dunphy et al., 2018; Luksch et al., 2019; Chu et al., 2021). Therefore, this study sought to determine the effects of cGAS deficiency and to identify a potential DAMP that may influence the induction of the canonical cGAS-STING pathway in CCI injury. Our data shows that *cGAS^−/−^* mice display significant neuroprotection and a blunted ISG response, similar to *STING*^−/−^ mice. This correlates with the observation of increased cytoplasmic mtDNA in the damaged cortex and suggests mtDNA is a possible DAMP that induces the cGAS-STING pathway in microglia leading to Type I interferon-induced tissue damage in TBI. Moreover, we demonstrate that NLRX1 is a novel upstream regulator of STING in this response.

Neuroinflammation is a critical component of the secondary injury response in TBI and offers a number of potential therapeutic targets, but is highly complex and remains poorly understood (Jassam et al., 2017). Our study selected a panel of genes associated with the Type I interferon response, including pro-inflammatory (*Il6*), anti-inflammatory (*Il10*), and pro-immune migratory cytokines (*MCP-1*), as well as transcription factors (*STAT1, STAT2,* and *IRF7*), interferons (*IFNA4* and *IFNB1*), and ISGs (*CXCL10, IFIT1, IFIT3,* and *IFIH1*). We determined that loss of cGAS or STING resulted in a broadly blunted immune response 24 h after injury. The decreased production of pro-inflammatory cytokines may contribute to the reduction in cell death seen in *cGAS*^−/−^ and *STING*^−/−^ animals (Figure 3). For example, work on stroke has shown that MCP1 deficiency reduces infarct size (Hughes et al., 2002) and BBB breakdown (Strecker et al., 2013), while MCP1 overexpression exacerbates tissue damage (Chen et al., 2003). Proteins in the JAK-STAT pathway, particularly STAT1, are also known to increase apoptosis both in the brain and periphery (Stephanou et al., 2000; Takagi et al., 2002; Nicolas et al., 2013).

Recent work has suggested that STING simultaneously stimulates the production of pro- and anti-inflammatory cytokines to facilitate the maintenance of gut homeostasis (Ahn et al., 2017), and studies in mouse models of systemic lupus erythematosus (SLE) have indicated STING signaling can be pro- or anti-inflammatory depending on the model (Sharma et al., 2015; Thim-uam et al., 2020; Motwani et al., 2021). Still, the autoimmune syndrome SAVI, caused by gain-of-function mutations in STING, results in excessive inflammation, indicating a primarily pro-inflammatory role for STING (Liu et al., 2014). Our data show altered mRNA expression of both pro- and anti-inflammatory cytokines in *cGAS^−/−^* and *STING*^−/−^ mice, suggesting that the effects of cGAS-STING signaling are highly complex and likely context-dependent. Further, the unselective upregulation of mRNAs for proteins with predominantly antiviral roles, such as *IFIT1* and *IFIT3*, suggests that this innate immune pathway is activated aberrantly after injury, unlike its normal role in viral or bacterial clearance. Further investigation is needed to clarify how the balance of pro- and anti-inflammatory cytokines are disrupted or skewed by alterations in cGAS-STING activity.

Recent findings show that mRNA expression of *STING* and key ISGs are elevated up to 60 days after experimental TBI (Barrett et al., 2020), indicating that STING activity may also contribute to chronic neuroinflammation. Consistently, we found that *cGAS*^−/−^ mice showed reduced motor deficits at 4 dpi and reduced lesion volume up to 14 dpi. Interestingly, we found that the Type I interferon ISG response was significantly reduced by 4 dpi (data not shown), and entirely resolved at 14 days (Supplementary Figure 2E). These data suggest that while the cGAS-STING signaling axis is acutely activated after injury, additional subsequent mechanisms may further contribute to the chronic progression of injury after trauma (McKee and Lukens, 2016). Further work is needed to define the temporal dynamics of cGAS-STING signaling after TBI.

It has been well-established that biological sex affects how the immune system responds to insult. Generally, females have a more robust immune response. Females produce more antibodies in response to H1N1 vaccination (Fink et al., 2018) and are at greater risk for autoimmune disease (Angum et al., 2020). However, how biological sex impacts TBI recovery appears highly dependent upon the model, outcome measure, and time points examined after injury (Berry et al., 2009; Späni et al., 2018; Gupte et al., 2019). While the incidence of TBI is significantly higher in males than females (Späni et al., 2018), we did not include female animal data in this study, and future studies will need to determine whether female animals show the same benefit with the loss of cGAS-STING signaling.

Conflicting evidence exists regarding whether NF-κB signaling is a major pathway activated downstream of STING (Stetson and Medzhitov, 2006; Zhong et al., 2008; Tanaka and Chen, 2012; Abe and Barber, 2014). However, recent work in mice with a point mutation in STING (S365A) that interfered with IRF3 binding elucidated that the switch between NF-κ signaling and Type I interferon signaling was context-dependent (Yum et al., 2021). With the generation of STING point mutation mouse models, future work could further define the contribution of different downstream effects of STING during TBI (Yum et al., 2021). Yet, Type-1 IFN receptor (IFNAR1) knockout mice are protected from TBI injury (Karve et al., 2016), indicating that the interferon pathway is still a major contributor to neuroinflammation in TBI. Future work is needed to elucidate cell-type- specific effects mediating the IFN response to TBI.

While our findings demonstrate the presence of cytosolic mtDNA in the injured cortex, it remains unclear how it is released into the cytosol after injury. Recently, the DNA/RNA binding protein TDP-43 has been implicated in the release of mtDNA *via* the mitochondrial permeability transition pore (mPTP) and subsequently caused cGAS-STING activation in a mouse model of ALS (Yu et al., 2020). Other work has shown that BAK/BAX macropores facilitate mitochondrial herniation and mtDNA efflux independent of the mPTP (McArthur et al., 2018; Riley et al., 2018). Mechanical forces have also been shown to promote mitochondrial fission events (Helle et al., 2017) that may allow for mtDNA release; indeed, TBI is associated with increases in mitochondrial fission and the fission-initiating dynamin-related protein 1 (Drp1; Fischer et al., 2016). Clarifying how mtDNA is released following neurotrauma may offer alternative therapeutic targets for reducing cGAS-STING-mediated neuroinflammation. Additionally, it remains possible that nuclear DNA is present in the cytosol. Further study will be required to determine whether nuclear DNA is or is not responsible for the activation of this pathway in neurotrauma.

The development of commercially-available small molecule STING antagonists provides the possibility for *in vivo* assessment of pharmaceutical inhibition of this pathway in the near future. Indeed, a few of these molecules have already shown promise in preventing viral infection tissue damage and autoinflammatory disease in mouse models (Haag et al., 2018; Domizio et al., 2022). Future work should evaluate whether these molecules may be beneficial for TBI, including evaluating their ability to cross the BBB.

Taken together, these data confirm that STING-mediated IFN signaling is detrimental to TBI-induced tissue damage. We have shown that loss of cGAS or STING results in improved histological and functional measures up to 14 days after TBI. Additionally, we provide evidence that NLRX1 negatively regulates STING activation in the brain, offering an additional potential target for therapeutic intervention. Perhaps most significantly, this study is the first to investigate mtDNA as a possible trigger for STING-IFN signaling in neurotrauma. Overall, our findings indicate that the canonical cGAS-STING-mediated ISG response is an early neuroinflammatory event occurring after cortical trauma, which represents a novel therapeutic target for treatment.

## Data Availability Statement

The raw data supporting the conclusions of this article will be made available by the authors, without undue reservation.

## Ethics Statement

The animal study was reviewed and approved by the Virginia Tech Institutional Animal Care and Use Committee.

## Author Contributions

LF wrote the first draft, performed experiments, and analyzed data. JJ, EG, ES, SP, AK, EK, JC, TT, RS, and XW performed experiments and analyzed data. IA provided the *Nlrx1^−/−^* mice and analyzed data. MT designed the project, analyzed data, and edited the manuscript. AP designed the project, performed experiments, analyzed data, and wrote the manuscript. All authors contributed to the article and approved the submitted version.

## Conflict of Interest

The authors declare that the research was conducted in the absence of any commercial or financial relationships that could be construed as a potential conflict of interest.

## Publisher’s Note

All claims expressed in this article are solely those of the authors and do not necessarily represent those of their affiliated organizations, or those of the publisher, the editors and the reviewers. Any product that may be evaluated in this article, or claim that may be made by its manufacturer, is not guaranteed or endorsed by the publisher.

## Supplementary Materials

Supplementary Figure S1 Western blotting to confirm cGAS and STING KO. **(A,B)** Representative western blot for STING and cGAS protein in brain homogenates from WT, *STING^−/−^*, and *cGAS*^−/−^ mice. Each lane represents an individual animal.

Supplementary Figure S2 cGAS^−/−^ mice show reduced motor deficit after TBI. Rotarod performance compared to baseline for cGAS^−/−^ and WT animals 4–14 days following sham **(A)** or CCI **(B)** surgery. *n* = 5 per genotype for **(A)** and *n* = 15 per genotype for **(B)**. **(C)** Lesion volume of WT, *STING*^−/−^, and *cGAS*^−/−^ brains 14 dpi. **(D)** Representative Cresyl violet stained WT, STING^−/−^, and cGAS^−/−^ brains 14 dpi. Dashed lines indicate lesion site. Scale bar = 1 mm. **(E)** mRNA expression of *IFNA4, IFNB1* and *Il6* assessed *via* qPCR 14 dpi or sham surgery for WT and cGAS KO animals. *n* = 5–7 per group. Data presented as mean ± SEM. Two-way ANOVA used for **(A)** and **(B)**, one-way ANOVA for **(C)** and **(E)**. **p* < 0.05, ***p* < 0.01, ****p* < 0.001, *****p* < 0.0001.

Supplementary Figure S3 Comparison of cytokine expression between ipsilateral sham and contralateral injured tissue. mRNA expression of **(A)** *Il6* and **(B)** *Il10* 2 h after surgery from the cortices of sham and CCI-injured WT animals. Gene expression was normalized to GAPDH. *n* = 5–6 per group. Data presented as mean ± SEM.

Supplementary Figure S4 Loss of STING attenuates cytokine and ISG response after injury. **(A–C)** Cytokine and interferon-stimulated gene (ISG) expression profiled 24 h after CCI from the contralateral and ipsilateral hemispheres of STING^−/−^ and WT mice. Cortical expression of **(A)** *CXCL10*, **(B)** *IRF7*, **(C)** *IFIT1*, **(D)** *IFIT3*, **(E)** *STAT1*, **(F)** *STAT2*, **(G)** *RIG-I*, and **(H)** *IFIH1* in WT and STING KO animals 24 h post-TBI. Gene expression was normalized to GAPDH. *n* = 5–6 per group. Data presented as mean ± SEM. **p* < 0.05, ***p* < 0.01, ****p* < 0.001, *****p* < 0.0001.

Supplementary Figure S5 Microglia upregulate cGAS and STING after injury. mRNA expression of **(A)** *cGAS* and **(B)** *STING* in isolated microglia collected 2- and 24 h after CCI or sham surgery. **(C)** mRNA expression of *STING* in non-adherent (non-microglia) cells remaining after microglia isolation 2- and 24 h after CCI or sham surgery. Gene expression was normalized to GAPDH and is relative to sham for each timepoint. *n* = 3–4 per group with pooled pairs of mice representing an n. Data presented as mean ± SEM. ***p* < 0.01.

## Abbreviations

BBB: blood brain barrier
CCI: controlled cortical impact
CDN: cyclic dinucleotide
cGAMP: cyclic guanosine monophosphate-adenosine monophosphate
cGAS: cyclic GMP-AMP synthase
CNS: central nervous system
CXCL10: C-X-C motif chemokine ligand 10
DAMP: damage-associated molecular pattern
Drp1: dynamin-related protein 1
GAPDH: glyceraldehyde 3-phosphate dehydrogenase
IFIH: interferon-induced helicase C domain-containing protein
IFIT: interferon-induced proteins with tetratricopeptide repeats
IFN: interferon
IFNAR: interferon alpha/beta receptor
IL: interleukin
IRF: interferon response factor
ISG: interferon-stimulated gene
JAK-STAT: Janus kinase-signal transducer and activator of transcription
KO: knock out
MCP-1: monocyte chemoattractant protein 1
MMP: matrix metalloproteinase
mPTP: mitochondrial permeability transition pore
mtDNA: mitochondrial DNA
NF-kB: nuclear factor kappa-light-chain-enhancer of activated B cells
NLRX1: NOD-like receptor containing X1
PAMP: pathogen-associated molecular pattern
PRR: pattern recognition receptor
qPCR: quantitative polymerase chain reaction
RIG-I: retinoic acid-inducible gene I
ROS: reactive oxygen species
SAVI: STING-associated vasculopathy with onset in infancy
SLE: systemic lupus erythematosus
STAT: signal transducer and activator of transcription
STING: stimulator of interferon genes
TBI: traumatic brain injury
TBK1: tank-binding kinase 1
TFAM: transcription factor A, mitochondrial
TLR: toll-like receptor
TUNEL: terminal deoxynucleotidyl transferase dUTP nick end labeling
VEGF: vascular endothelial growth factor
WT: wildtype.
