# Corrigendum: Type I Interferon Response Is Mediated by NLRX1-cGAS-STING Signaling in Brain Injury

**DOI:** 10.3389/fnmol.2022.965564

**Published:** 2022-06-29

**Authors:** Lauren E. Fritsch, Jing Ju, Erwin Kristobal Gudenschwager Basso, Eman Soliman, Swagatika Paul, Jiang Chen, Alexandra M. Kaloss, Elizabeth A. Kowalski, Taylor C. Tuhy, Rachana Deven Somaiya, Xia Wang, Irving Coy Allen, Michelle H. Theus, Alicia M. Pickrell

**Affiliations:** ^1^Translational Biology, Medicine, and Health Graduate Program, Virginia Polytechnic Institute and State University, Roanoke, VA, United States; ^2^Molecular and Cellular Biology Graduate Program, Virginia Polytechnic Institute and State University, Blacksburg, VA, United States; ^3^Department of Biomedical Sciences and Pathobiology, Virginia Polytechnic Institute and State University, Blacksburg, VA, United States; ^4^Biomedical and Veterinary Sciences Graduate Program, Virginia Polytechnic Institute and State University, Blacksburg, VA, United States; ^5^School of Neuroscience, Virginia Polytechnic Institute and State University, Blacksburg, VA, United States

**Keywords:** brain injury, inflammation, STING, cGAS, innate immunity

In the published article, there was an error in Table 1 as published. The forward and reverse sequences for the rows “IL-6” and “MCP1” were incorrectly entered as “CTA GCT CAG GCT CGT CAG TTC.” The corrected Table 1 and its caption appear below.

**Table 1:** qPCR primers used in experiments.

**Gene**	**Forward Seq. (5^**′**^-3^**′**^)**	**Reverse Seq. (5^**′**^-3^**′**^)**
IRF7	CAA TTC AGG GGA TCC AGT TG	AGC ATT GCT GAG GCT CAC TT
IFIT1	ACC ATG GGA GAG AAT GCT GAT G	TGT GCA TCC CCA ATG GGT TC
STAT1	GCG GCA TGC AAC TGG CAT ATA ACT	ATG CTT CCG TTC CCA CGT AGA CTT
STAT2	TGA TCT CTA ACA GAC AGG TGG	CTG CAT TCA CTT CTA AAG ACT C
IFIT3	ATC ATG ATG GAG GTC AAC CG	TTG CAC ACC CTG TCT TCC AT
IFNA4	CTT TCC TCA TGA TCC TGG TAA TGA T	AAT CCA AAA TCC TTC CTG TCC TCC
IFNB1	AAC TCC ACC AGC AGA CAG TG	GGT ACC TTT GCA CCC TCC AG
RIG-I	GAG TAC CAC TTA AAG CCA GAG	AAT CCA TTT CTT CAG AGC ATC C
IFIH1	CGG AAG TTG GAG TCA AAG C	TTT GTT CAG TCT GAG TCA TGG
IL-10	AGA CCA AGG TGT CTA CAA GGC	TCA TCA TGT ATG CTT CTA TGC AGT
IL-6	ACA AGT CGG AGG CTT AAT TAC ACA	TTG CCA TTG CAC AAC TCT TTT C
MCP1	TCA CCT GCT GCT ACT CAT TCA CCA	TAC AGC TTC TTT GGG ACA CCT GCT
CXCL10	ATA ACC CCT TGG GAA GAT GGT G	CTA GCT CAG GCT CGT CAG TTC
GAPDH	ATT GTG TCC GTC GTG GAT CTG A	AGA TGC CTG CTT CAC CAC CTT CTT
STING	GCC TTC AGA GCT TGA CTC CA	GTA CAG TCT TCG GCT CCC TG
ND1	CAG CCT GAC CCA TAG CCA TA	ATT CTC CTT CTG TCA GGT CGA A
COX1	AGG CTT CAC CCT AGA TGA CAC	GTA GCG TCG TGG TAT TCC TGA A

*List of forward and reverse sequences used for qPCR*.

The authors apologize for this error and state that this does not change the scientific conclusions of the article in any way. The original article has been updated.

## Publisher's Note

All claims expressed in this article are solely those of the authors and do not necessarily represent those of their affiliated organizations, or those of the publisher, the editors and the reviewers. Any product that may be evaluated in this article, or claim that may be made by its manufacturer, is not guaranteed or endorsed by the publisher.

