# Supplementary figures and images for: Type I Interferon Response Is Mediated by NLRX1-cGAS-STING Signaling in Brain Injury
Source: Front Mol Neurosci. 2022 Feb 25;15:852243. doi: 10.3389/fnmol.2022.852243 (PMC8916033; doi:10.3389/fnmol.2022.852243)

# Supplemental Figure 1

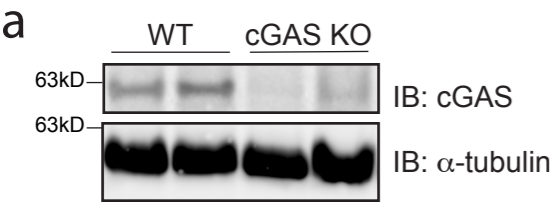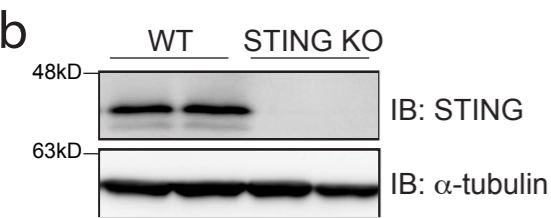

Supplement: Supplementary Figure S1 — Western blotting to confirm cGAS and STING KO. (A,B) Representative western blot for STING and cGAS protein in brain homogenates from WT, STING−/−, and cGAS−/− mice. Each lane represents an individual animal. [file Image_1.PDF]

Supplemental Figure 2

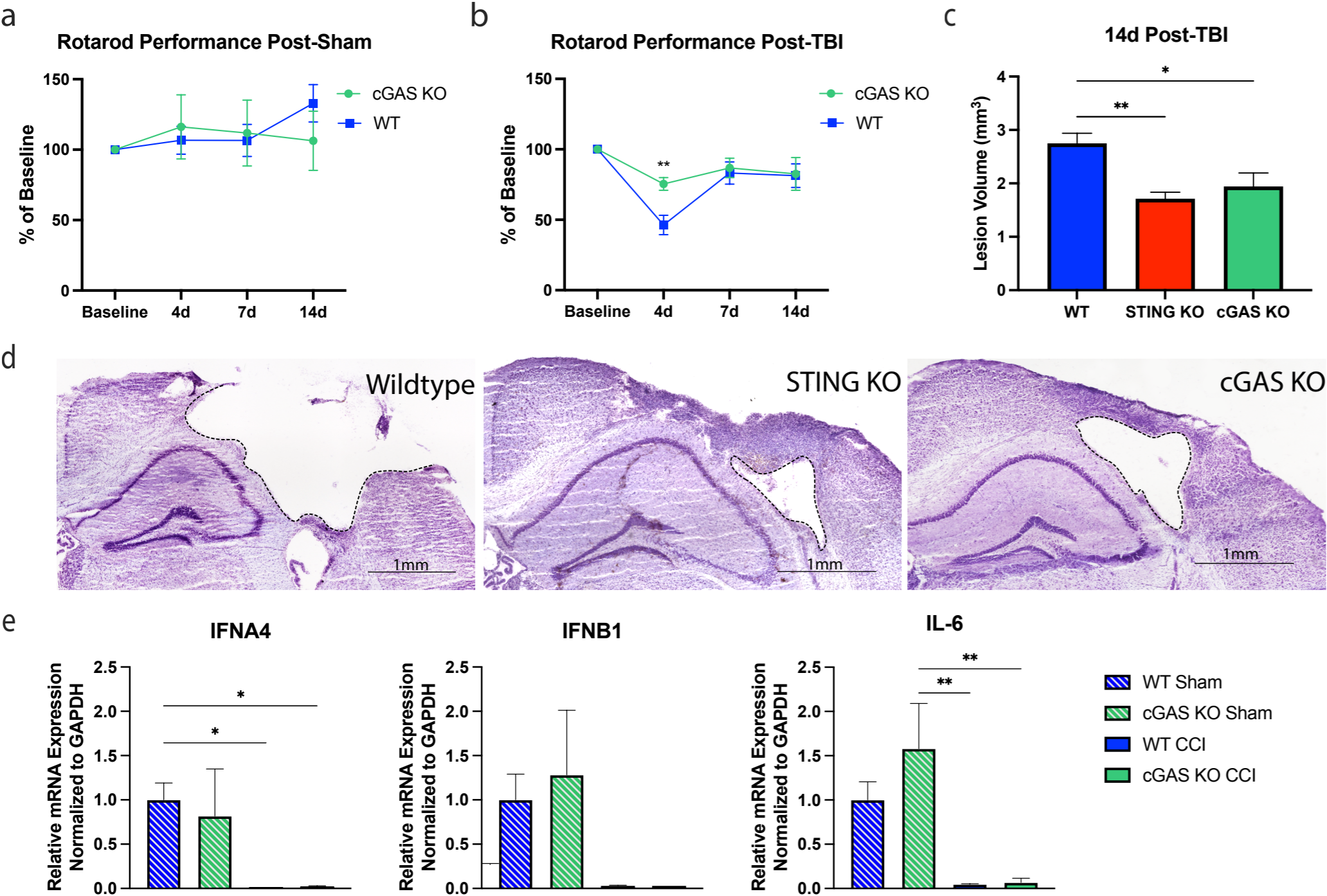

Supplement: Supplementary Figure S2 — cGAS−/− mice show reduced motor deficit after TBI. Rotarod performance compared to baseline for cGAS−/− and WT animals 4–14 days following sham (A) or CCI (B) surgery. n = 5 per genotype for (A) and n = 15 per genotype for (B). (C) Lesion volume of WT, STING−/−, and cGAS−/− brains 14 dpi. (D) Representative Cresyl violet stained WT, STING−/−, and cGAS−/− brains 14 dpi. Dashed lines indicate lesion site. Scale bar = 1 mm. (E) mRNA expression of IFNA4, IFNB1 and Il6 assessed via qPCR 14 dpi or sham surgery for WT and cGAS KO animals. n = 5–7 per group. Data presented as mean ± SEM. Two-way ANOVA used for (A) and (B), one-way ANOVA for (C) and (E). *p < 0.05, **p < 0.01, ***p < 0.001, ****p < 0.0001. [file Image_2.PDF]

Supplemental Figure 3

a

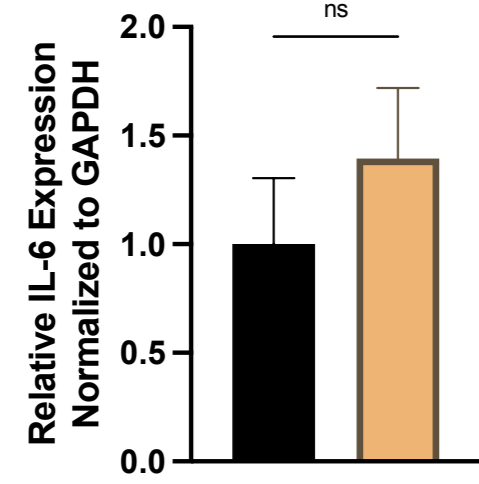

b

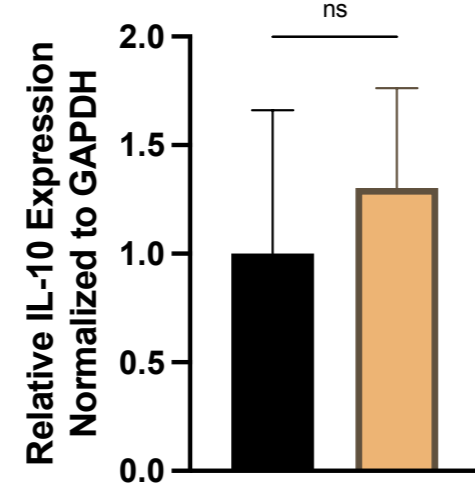

CCI Contralateral  
Sham Ipsilateral

Supplement: Supplementary Figure S3 — Comparison of cytokine expression between ipsilateral sham and contralateral injured tissue. mRNA expression of (A) Il6 and (B) Il10 2 h after surgery from the cortices of sham and CCI-injured WT animals. Gene expression was normalized to GAPDH. n = 5–6 per group. Data presented as mean ± SEM. [file Image_3.PDF]

Supplemental Figure 4

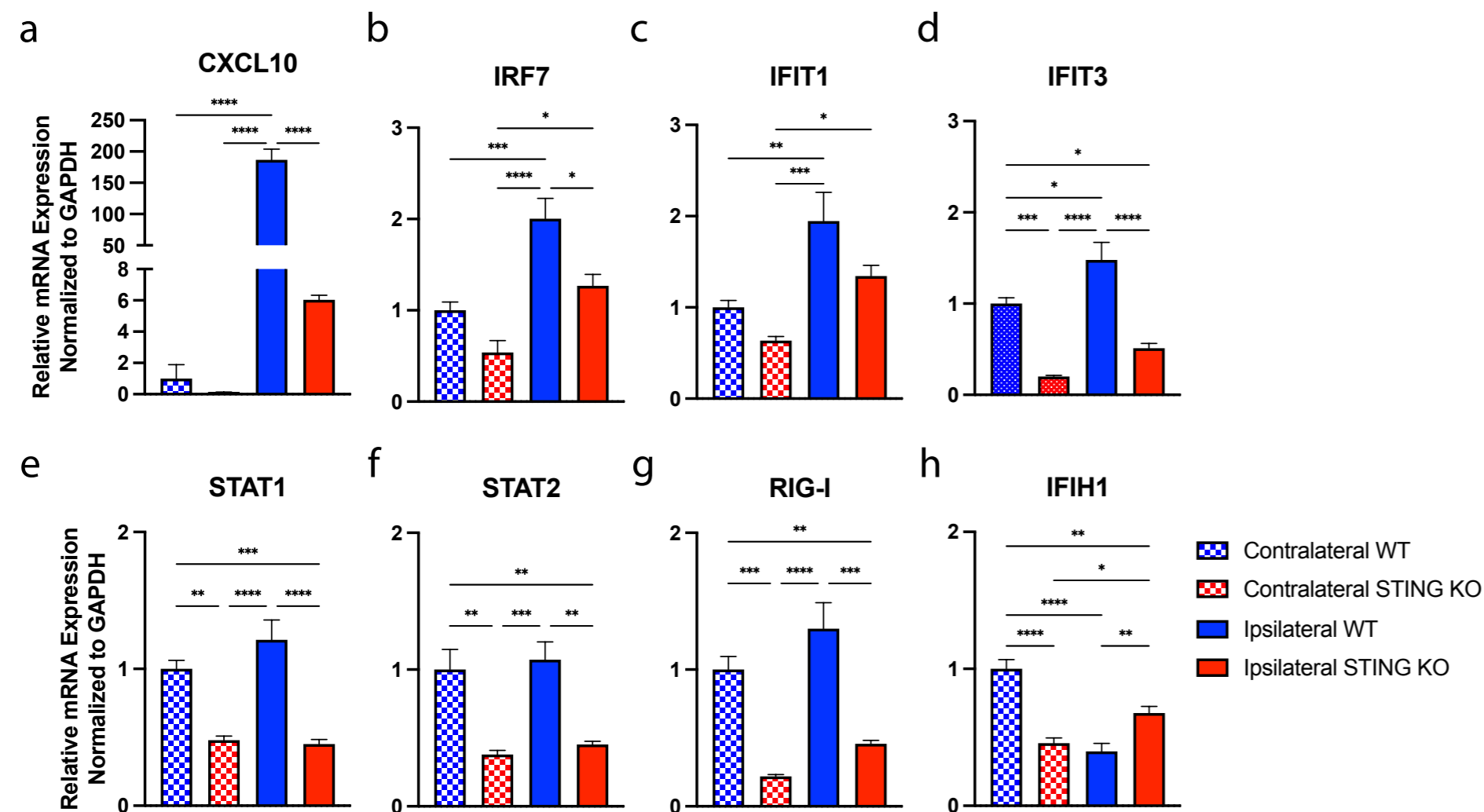

Supplement: Supplementary Figure S4 — Loss of STING attenuates cytokine and ISG response after injury. (A–C) Cytokine and interferon-stimulated gene (ISG) expression profiled 24 h after CCI from the contralateral and ipsilateral hemispheres of STING−/− and WT mice. Cortical expression of (A) CXCL10, (B) IRF7, (C) IFIT1, (D) IFIT3, (E) STAT1, (F) STAT2, (G) RIG-I, and (H) IFIH1 in WT and STING KO animals 24 h post-TBI. Gene expression was normalized to GAPDH. n = 5–6 per group. Data presented as mean ± SEM. *p < 0.05, **p < 0.01, ***p < 0.001, ****p < 0.0001. [file Image_4.PDF]

Supplemental Figure 5

a

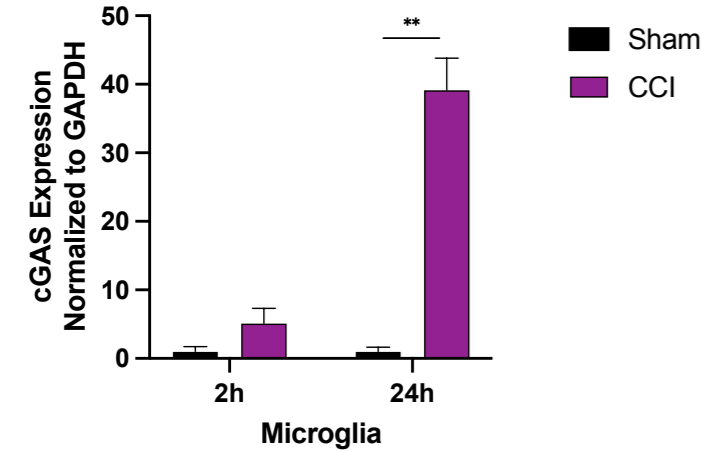

b

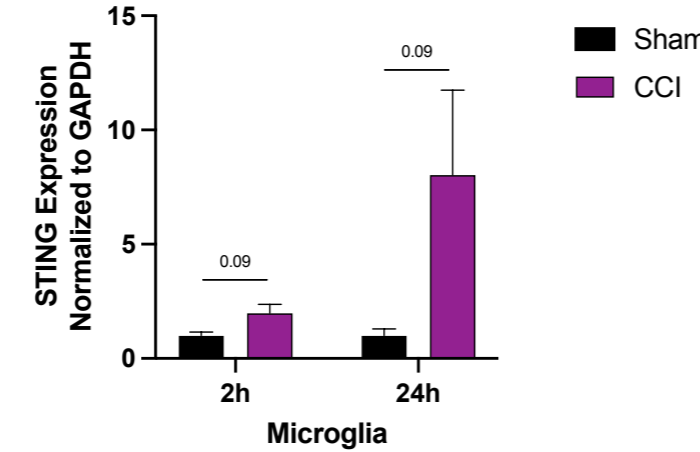

c

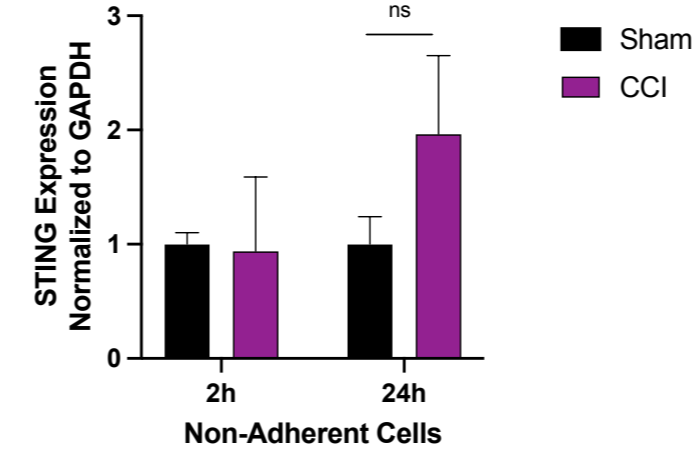

Supplement: Supplementary Figure S5 — Microglia upregulate cGAS and STING after injury. mRNA expression of (A) cGAS and (B) STING in isolated microglia collected 2- and 24 h after CCI or sham surgery. (C) mRNA expression of STING in non-adherent (non-microglia) cells remaining after microglia isolation 2- and 24 h after CCI or sham surgery. Gene expression was normalized to GAPDH and is relative to sham for each timepoint. n = 3–4 per group with pooled pairs of mice representing an n. Data presented as mean ± SEM. **p < 0.01. [file Image_5.PDF]
